# Research on the control strategy of distributed energy resources inverter based on improved virtual synchronous generator

**DOI:** 10.1038/s41598-017-09787-w

**Published:** 2017-08-22

**Authors:** Changwei Gao, Xiaoming Liu, Hai Chen

**Affiliations:** 1 School of Electrical Engineering, Shenyang University of Technology, Shenyang, CO 110870 China; 2 College of Electrical and Information Engineering, Liaoning Institute of Since and Technology, Benxi, CO 117004 China; 3 College of Electrical Engineering and Automation, Tianjin Polytechnic University, Tianjin, CO 300387 China

## Abstract

This paper focus on the power fluctuations of the virtual synchronous generator(VSG) during the transition process. An improved virtual synchronous generator(IVSG) control strategy based on feed-forward compensation is proposed. Adjustable parameter of the compensation section can be modified to achieve the goal of reducing the order of the system. It can effectively suppress the power fluctuations of the VSG in transient process. To verify the effectiveness of the proposed control strategy for distributed energy resources inverter, the simulation model is set up in MATLAB/SIMULINK platform and physical experiment platform is established. Simulation and experiment results demonstrate the effectiveness of the proposed IVSG control strategy.

## Introduction

Conventional fossil energy has been unable to meet the needs of sustainable development of human society. The advantages for distributed energy resources are of flexibility, no pollution, and wide distribution. It becomes suitable energy that conforms to the concept of sustainable development^1, 2^. In many fields, it is changing from complementary energy to alternative energy gradually^3, 4^. In conventional electrical power system, the power is provided by the synchronous generator basically. Synchronous generator has the mechanical inertia. To maintain the dynamic stability of the power system, the synchronous generator makes energy exchange with power system by means of the kinetic energy stored in the rotor when disturbance occurs. Distributed energy resources is connected to the power system via the power electronic converter, and it almost doesn’t have inertia and damping. Because of this disadvantage, the distributed energy resources system is not conducive to the frequency modulation and voltage regulation of the power system^5, 6^. It has a negative effect on the dynamic response and stability of the power system^7–11^.

In order to make the inverter of a distributed generating unit present the characteristics similar to synchronous generator, in recent years, virtual synchronous generator(VSG) control technology based on the synchronous generator electromechanical transient analysis theory has received the widespread attention of scholars around the world. Literature^12^ put forward the concept of VSG for the first time, synchronous generator model was used to control the output current of the inverter of a distributed generating unit. Literature^13^ proposed a voltage source type VSG control strategy, which can operates in two modes of interconnection and autonomy. It further improves the frequency stability of the system. Literature^14^ introduced the partial linear model of the synchronous generator into the conventional active-frequency droop control. On the basis of the conventional droop control, it effectively simulated the synchronous generator’s rotation inertia, damping, and the characteristics of primary frequency control. Literatures^15–18^ made a deep research on the rotor inertia adaptive control strategy of the virtual synchronous generator, and it has greatly improved the dynamic response speed of the conventional virtual synchronous generator. In previous research^19^, a virtual mechanical phase is established using the swing equation with simple damping term. Then it was verified that VSG system enhance the grid stability. A virtual synchronous generator with the functions of P-Q control and V-f control in microgrid is proposed in ref. 20. The core algorithm of VSG is to employ an electromechanical transient mathematic model of round SG. In previous research^21^, a reactive power control method was developed to keep the sending end voltage at the specified rated value and operate with the same control scheme whenever it is connected to the grid or operated in intentional islanding mode. In addition, many other literatures have carried on the different research. Compared with conventional droop control strategy, the results show that the VSG has a great advantage^22–25^.

However, virtual synchronous generator(VSG) have active and reactive power fluctuations in transient process. A novel linearizing technique for VSG-based control scheme is proposed in ref. 26. Thus, the VSG can damp the grid oscillations by linear control theory. On the basis of the previous research, a control strategy based on improved virtual synchronous generator(IVSG) is proposed in this paper. Mathematical model of the virtual synchronous generator is studied, and the effects of rotational inertia in transient process are analyzed. The IVSG control method based on feed-forward compensation is therefore proposed. Adjustable parameter of the compensation unit can be modified to achieve the goal of reducing system order. It can restrain the active power fluctuation effectively.

## IVSG control strategy

### VSG control principle

**Figure 1:**
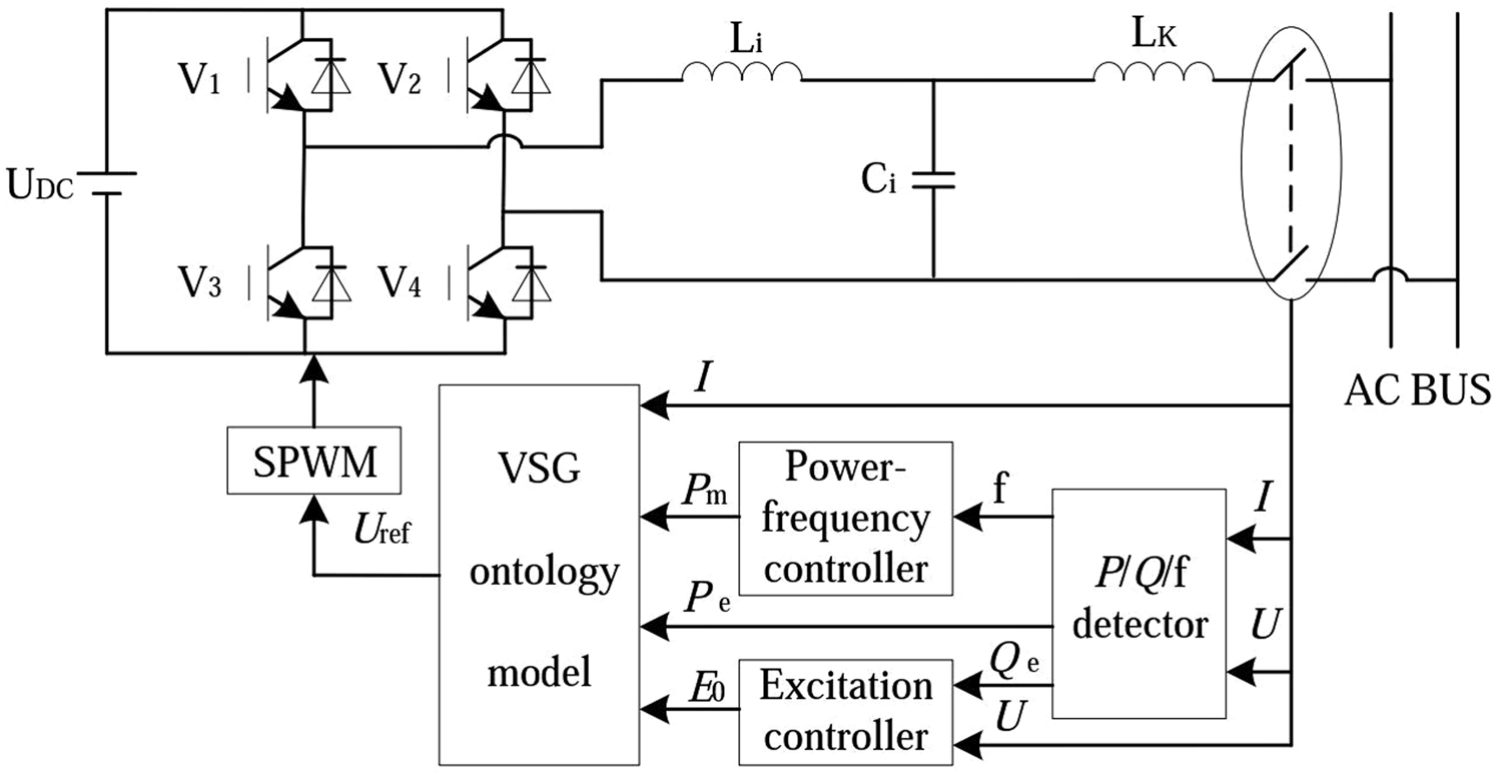
The structure of VSG.

The structure of VSG is shown in Fig. 1. Control system collects parameters from AC bus entrance. Electromagnetic power(*P*_*e*_), reactive power(*Q*_e_) and frequency(f) are calculated by the *P*/*Q*/f detector. Mechanical power (*P*_*m*_) is collected by the power-frequency controller, and excitation voltage (*E*_0_) is collected by the excitation controller respectively. The signals across the VSG algorithm model are converted to reference voltage (*U*_*ref*_). The output voltage of the inverter via LC filter circuit creates the grid connected voltage. In this way, the distributed inverter has the characteristics that are similar to the synchronous generator.

### VSG mathematical model analysis

Second-order model is widely applied in the VSG ontology control strategy. The mathematical model of synchronous generator is expressed as follows:

1 $$\{\begin{array}{c}{M}_{m}-{M}_{e}-D{\rm{\Delta }}\omega =\frac{{P}_{m}}{\omega }-\frac{{P}_{e}}{\omega }-D{\rm{\Delta }}\omega =J\frac{d{\rm{\Delta }}\omega }{dt}\\ \frac{d\theta }{dt}=\omega \\ \mathop{E}\limits^{\bullet }=\mathop{U}\limits^{\bullet }+\mathop{I}\limits^{\bullet }(r+jx)\end{array}$$

where *M*_*m*_ is mechanical torque, *M*_*e*_ is electromagnetic torque, *P*_*m*_ is mechanical power, *P*_*e*_ is electromagnetic power, *D* is damping factor, *Δω* is the difference of electrical angular velocity (Δ*ω = ω-ω*_0_, *ω*_0_ is the rated electrical angular velocity, *ω* is the actual value of electrical angular velocity), *J* is the rotational inertia of the rotor, *θ* is electric angle, E is induction electromotive force, *U* is the terminal voltage of the stator, *I* is stator current, *r* is the armature resistance of the stator, and *x* is synchronous reactance.

**Figure 2:**
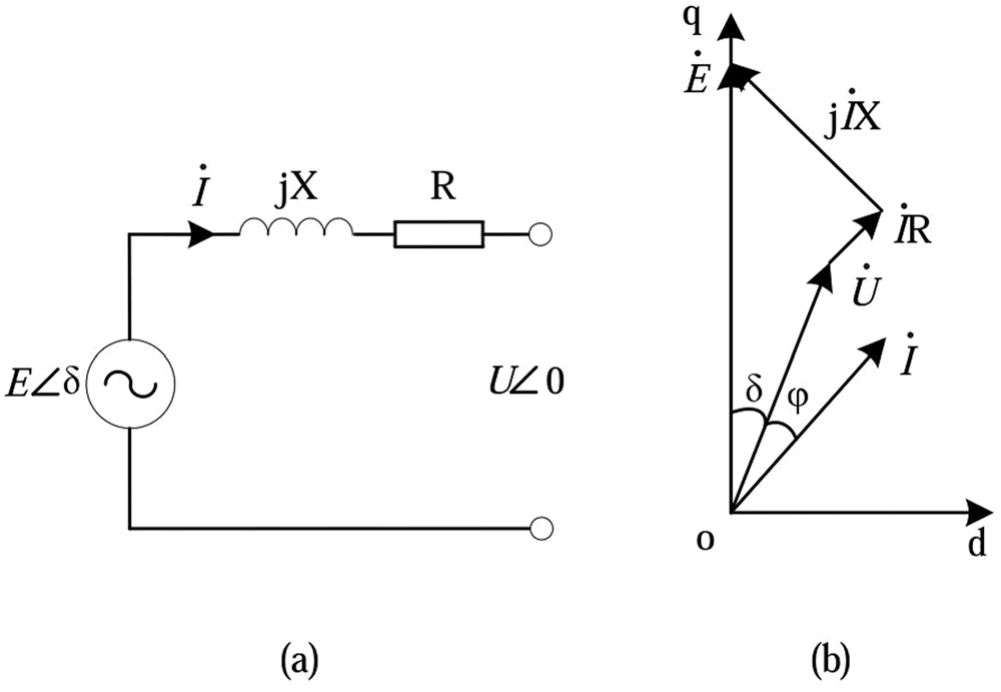
The equivalent circuit and the phasor diagram of VSG. (**a**) Equivalent circuit. (**b**) Phasor diagram.

Figure 2 shows the equivalent circuit and the phasor diagram of VSG. *E* and *U* represent the output voltage amplitude and the load voltage amplitude of the VSG respectively. *δ* is the phase difference between them. In this circuit, *Z* = *R* + *jX*, ‘Z’ is the sum of the output impedance of the inverter and the line impedance.

In Fig. 2(a), the loop-voltage equation is given as follows:

2 $$\mathop{E}\limits^{\bullet }=\mathop{U}\limits^{\bullet }+\mathop{I}\limits^{\bullet }R+j\mathop{I}\limits^{\bullet }X$$

Figure 2(b) shows the phasor diagram of the VSG. According to the definition, the complex power of the VSG can be expressed as follows:

3 $$\mathop{S}\limits^{ \sim }=\mathop{U}\limits^{\bullet }\mathop{I}\limits^{\ast }={P}_{e}+j{Q}_{e}=({U}_{d}+j{U}_{q})({I}_{d}-j{I}_{q})$$

where

4 $${P}_{e}={U}_{d}{I}_{d}+{U}_{q}{I}_{q}$$

5 $${Q}_{e}={U}_{q}{I}_{d}-{U}_{d}{I}_{q}$$

As shown in Fig. 2(b), *I*_*d*_ and *I*_*q*_ can be indicated as follows:

6 $${I}_{d}=\frac{E-{U}_{q}-{I}_{q}R}{X}$$

7 $${I}_{q}=\frac{{U}_{d}+{I}_{d}R}{X}$$

Eq. (4) and Eq. (5) may then be written as follows:

8 $${P}_{e}={U}_{d}\frac{E-{U}_{q}}{X}-{U}_{d}\frac{{I}_{q}R}{X}+{U}_{q}\frac{{U}_{d}}{X}+{U}_{q}\frac{{I}_{d}R}{X}=\frac{EU}{X}\,\sin \,\delta +\frac{R}{X}({U}_{q}{I}_{d}-{U}_{d}{I}_{q})$$

9 $${Q}_{e}={U}_{q}\frac{E-{U}_{q}-{I}_{q}R}{X}-{U}_{d}\frac{{U}_{d}+{I}_{d}R}{X}=\frac{EU}{X}\,\cos \,\delta -\frac{{U}^{2}}{X}-\frac{R}{X}({U}_{q}{I}_{q}+{U}_{d}{I}_{d})$$

Actually, power system has a lot of reactive compensation devices. Therefore, power factor is close to 1. In this case, as shown in Fig. 2(b), *U*_*q*_ *I*_*d*_ is approximately equal to *U*_*d*_ *I*_*q*_. Generally, *δ* is very small, Eq. (8) can be simplified as follows:

10 $${P}_{e}=\frac{EU}{X}\delta =K\delta $$

11 $$\delta =\int (\omega -{\omega }_{0})dt$$

On the basis of Eq. (10) and Eq. (11), the following equations can be derived:

12 $$\frac{d{P}_{e}}{dt}=K\frac{d\delta }{dt}=K(\omega -{\omega }_{0})$$

13 $$\frac{{d}^{2}{P}_{e}}{d{t}^{2}}=K\frac{d\omega }{dt}$$

Therefore, the rotor motion equation in Eq. (1) can be expressed as follows:

14 $${P}_{m}={P}_{e}+D\frac{d{P}_{e}}{dt}\frac{1}{K}+J\frac{{d}^{2}{P}_{e}}{{{\rm{dt}}}^{{\rm{2}}}}\frac{1}{K}$$

Then by using Laplace transform, Eq. (14) can be indicated as follows:

15 $$\frac{{P}_{e}}{{P}_{m}}=\frac{K/J}{{s}^{2}+Ds/J+K/J}$$

**Figure 3:**
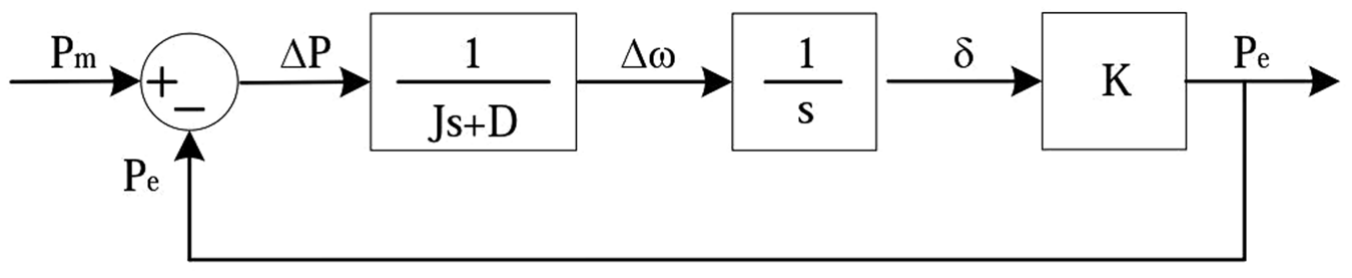
The closed-loop power control structure of the VSG.

Therefore, the closed-loop power control structure of the VSG can be shown as Fig. 3.

The closed loop transfer function of power-frequency fluctuations of the VSG can be written as follows:

16 $$\frac{\Delta P}{\Delta \omega }=-\frac{1}{s/K+1/(Js+D)}$$

**Figure 4:**
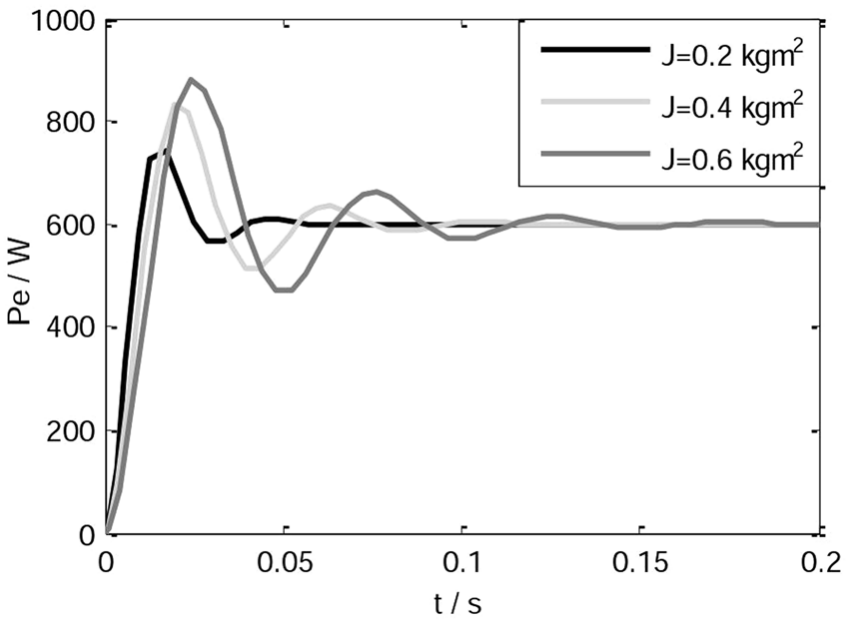
Active power responses of the VSG.

When the rotational inertia is set to different values, the active power responses of the VSG are shown in Fig. 4. As noted in Fig. 4, the bigger the rotational inertia is, the greater the overshoot of the system and the active power fluctuations are.

### IVSG control strategy

Because of the introduction of the rotational inertia *J*, Eq. (16) is the transfer function of a typical second order system. In order to overcome the power fluctuations of the VSG in transient process, the closed loop transfer function of power-frequency fluctuations is assumed to be the first order inertia unit. So, Eq. (16) can be indicated as follows:

17 $$\frac{{\rm{\Delta }}P}{{\rm{\Delta }}\omega }=-\frac{1}{s/(CK)+1/D}$$

where *C* is adjustable parameter. When the feed-forward compensation unit(*G*_*f*_) is introduced, Eq. (17) can be expressed as follows:

18 $$\frac{{\rm{\Delta }}P}{{\rm{\Delta }}\omega }=-\frac{1}{s/K+{G}_{f}/{\rm{D}}}$$

Synthesize Eq. (17) and Eq. (18), the feed-forward compensation unit(*G*_*f*_) can be derived as follows:

19 $${G}_{f}=1+\frac{(1-C)Ds}{CK}$$

Therefore, the closed loop transfer function of power-frequency fluctuations of the IVSG may then be written as follows:

20 $$\frac{{\rm{\Delta }}P}{{\rm{\Delta }}\omega }=-\frac{1}{s/K+[1+(1-C)Ds/CK]/(Js+D)}$$

**Figure 5:**
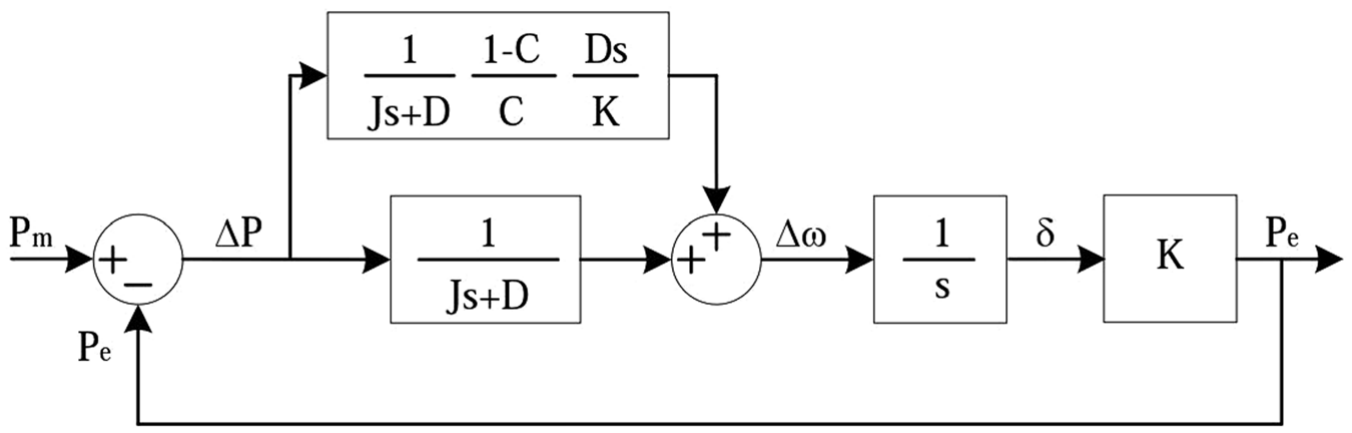
The closed-loop power control structure of the IVSG.

Closed-loop power control structure of the IVSG is shown as Fig. 5. When C is equal to 1, its control effect is the same as the conventional synchronous generator.

**Figure 6:**
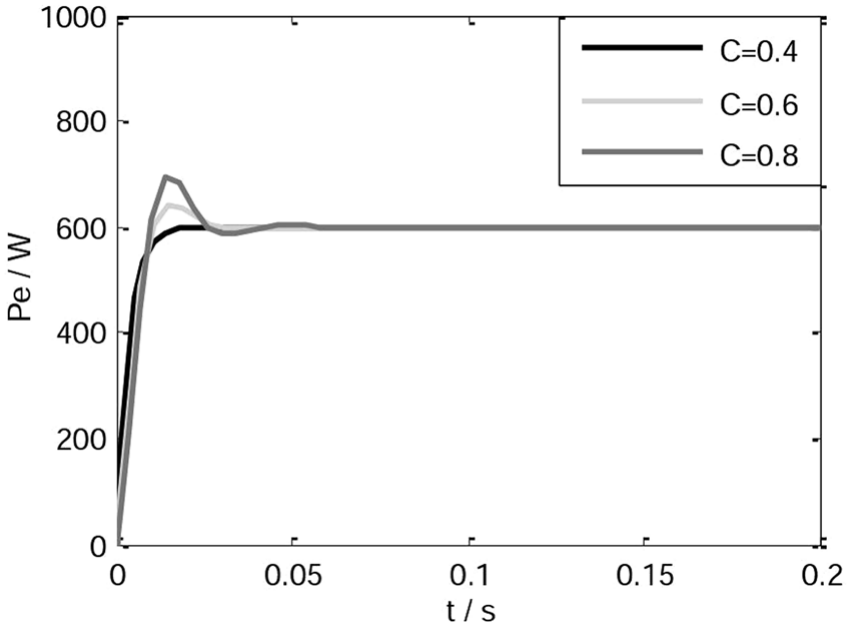
Active power responses under different adjusting parameters.

When adjustable parameter C is set to be different values, *J* = 0.2 kgm^2^, the active power responses are shown in Fig. 6. As shown in Fig. 6, the bigger the adjustable parameter is, the greater the overshoot of the system is. The appropriate reduction of the adjustable parameter C can avoid active power overshoot and speed up the response of the system.

### Power-frequency controller

**Figure 7:**
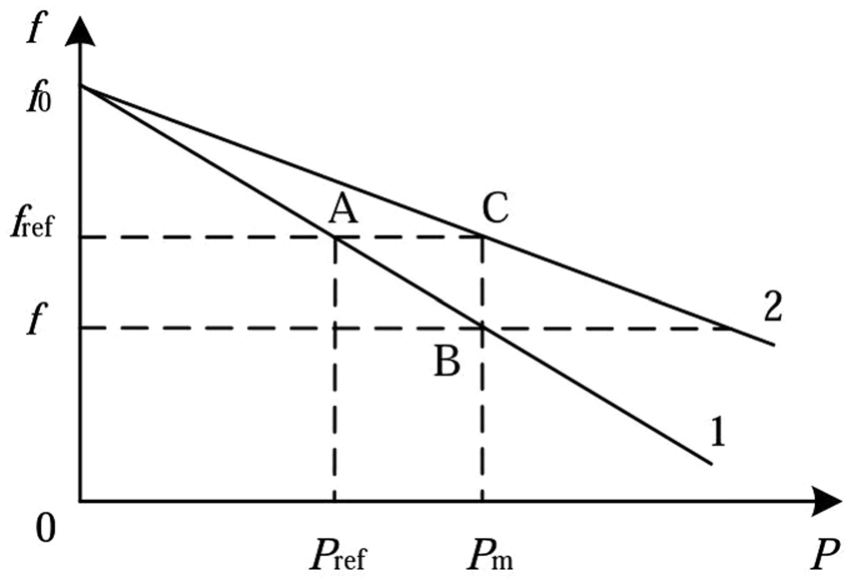
Power-frequency characteristic of frequency adaptive adjustment.

The frequency adaptive control strategy is proposed in this paper. The frequency modulation coefficient is adjusted with load change. Figure 7 is the power-frequency characteristic of frequency adaptive adjustment.

In Fig. 7, the initial running state of the system is at point A, the slope equation of the oblique line 1 is expressed as Eq. (21). *K*_*ω*_ is the slope of the oblique line 1 in the initial operation state.

21 $$\frac{1}{{K}_{\omega }}=\frac{{f}_{0}-{f}_{ref}}{{P}_{ref}}$$

The function of adaptive adjustment is to transform the oblique line 1 into the oblique line 2. Its physical meaning is that the output of active power in the process of adjustment is equal to that of the load, so the actual frequency can keep track of the frequency reference in real time. Eq. (22) is used to describe the slope equation of the oblique line 2 of adaptive adjustment. ${K}_{\omega }^{^{\prime} }$ is the slope.

22 $$\frac{1}{{K}_{\omega }^{^{\prime} }}=\frac{{f}_{0}-{f}_{ref}}{{P}_{m}}$$

Therefore, the relationship between *K*_*ω*_ and *K*_*ω*_^’^ is shown in equation (23).

23 $${K}_{\omega }^{\text{'}}=\frac{{P}_{e}}{{P}_{ref}}{K}_{\omega }$$

When adaptive adjustment strategy is adopted, the frequency control equation can be expressed as follows.

24 $$({f}_{0}-f){K}_{\omega }^{\text{'}}={P}_{m}$$

**Figure 8:**
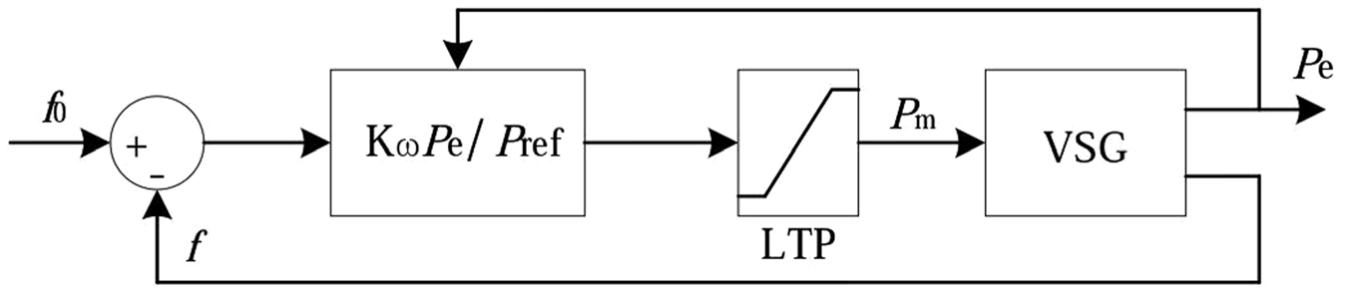
The structure of the power-frequency controller.

When adaptive adjustment strategy is adopted, the structure of the power-frequency controller is shown as Fig. 8. According to the Fig. 8 and above theoretical analysis, when the system runs at the initial point A, the output values of frequency and active power of the VSG are the frequency reference and active power reference respectively. When loads increase, the output power of VSG and the frequency modulation coefficient start to increase. Oblique line 1 becomes oblique line 2. When the output power of VSG is equal to the loads power, the system stability runs at point C, and the system frequency is still the frequency reference.

### The excitation controller

**Figure 9:**
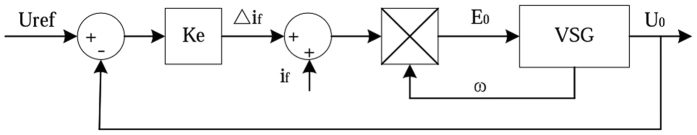
The structure of the excitation controller.

Considering the synchronous generator stator voltage equations and its excitation controller design principle, the structure of the excitation controller can be indicated as Fig. 9. *U*_*ref*_ is the reference of the output voltage amplitude, *U*_0_ is the actual output voltage amplitude, *K*_*e*_ is the proportion amplification coefficient. Δ*i*_*f*_ is the field current deviation, *i*_*f*_ is the reference of the field current, sum them and multiplied by angular frequency *ω* then results in the excitation electromotive force *E*_0_.

## Simulation analysis

**Figure 10:**
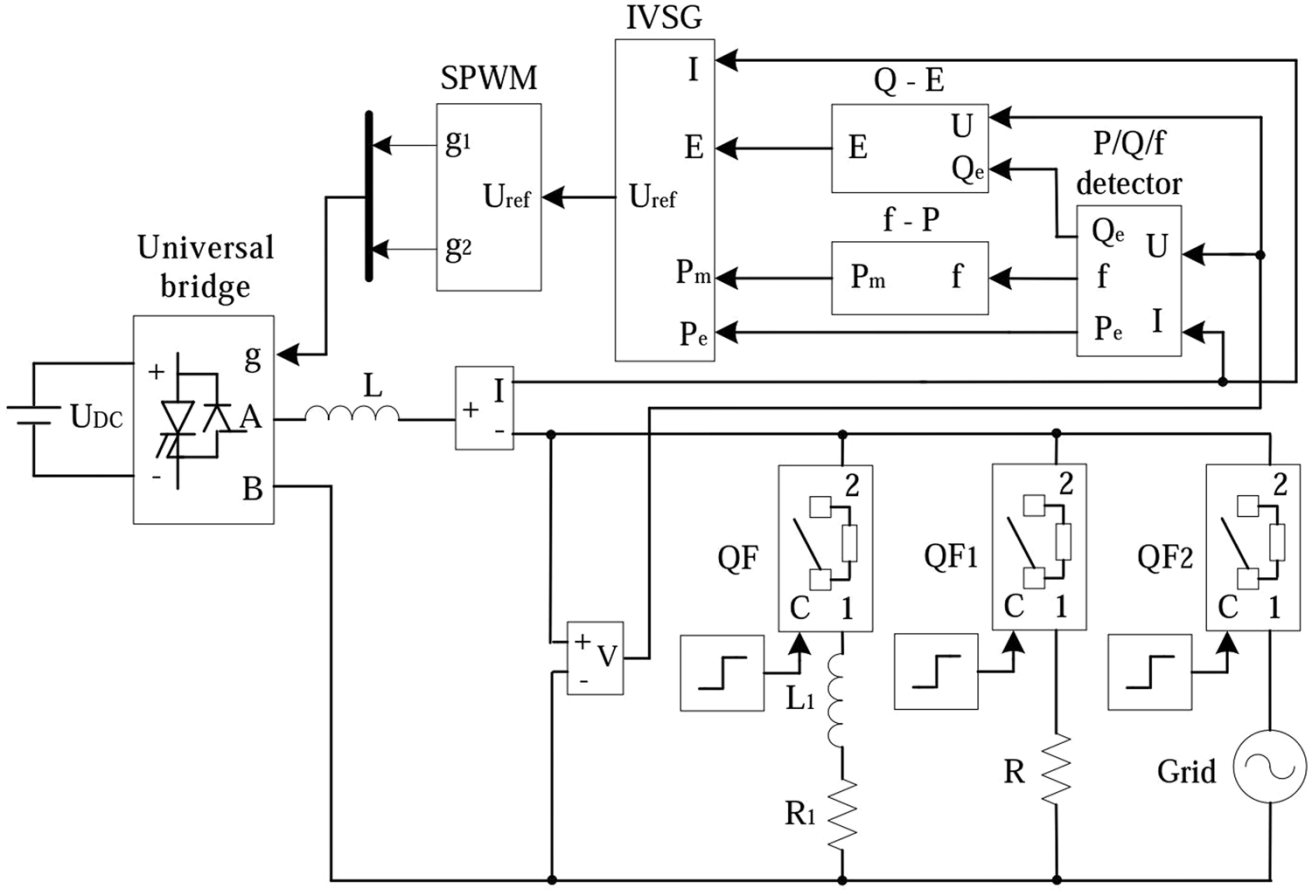
The simulation model of the IVSG.

According to the structure of Fig. 1, the simulation model is set up in MATLAB/SIMULINK platform to verify the effectiveness of the IVSG control strategy when it is applied to distributed generator. Figure 10 is the simulation model of the IVSG. As shown in Fig. 10, to simplify the complexity of the model, the DC voltage source is used to replace the distributed energy resources. IVSG module is the encapsulated subsystem of improved virtual synchronous generator ontology algorithm. SPWM module creates drive pulse for the inverter. Q-E module is the encapsulated subsystem of excitation controller, f-P module is the encapsulation of power- frequency controller. P/Q/f module is used to calculate electromagnetic power and frequency. QF, QF_1_ and QF_2_ are breakers. QF and QF_1_ control the change of the load. QF_2_ controls the grid-connection.

### Simulation analysis of isolated operation mode

The simulation time is set to 0.2 s. Initially, QF, QF_1_ and QF_2_ are turned off. When the simulation starts, QF is turned on, the series branch of R_1_ and L_1_ is put into operation. When the simulation time is 0.1 s, QF_1_ is turned on, and therefore loads increase. The series branch of R_1_ and L_1_ is used to simulate the inductance load. The branch of R is used to simulate the resistance load. *U*_*DC*_ = 400 V, R = R_1_ = 40 Ω, L_1_ = 10 mH.

**Figure 11:**
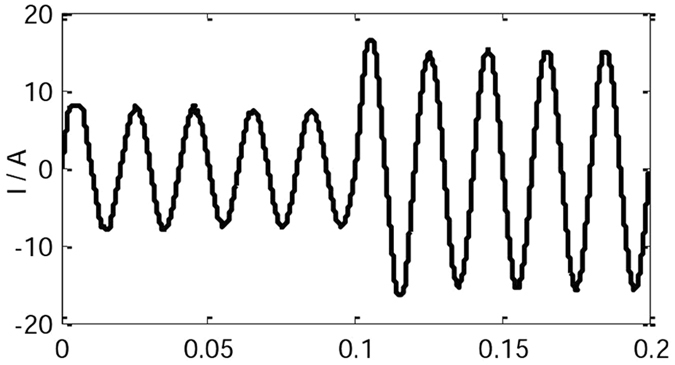
The current waveform of the conventional VSG.

**Figure 12:**
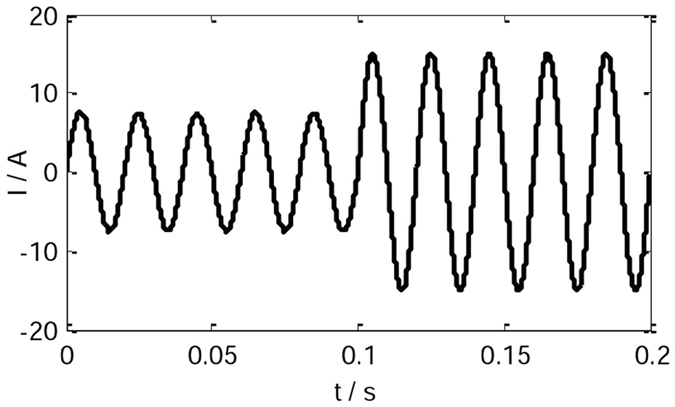
The current waveform of the IVSG.

Figure 11 is the current waveform of the conventional VSG control strategy(*J* = 0.2 kgm^2^). When the IVSG control strategy is adopted (*J* = 0.2 kgm^2^, *C* = 0.4), in the process of load change, the current waveform is shown in Fig. 12. As the figure shows, when the loads change, the shock current of the system is decreased significantly, and the stability of the system is improved greatly.

**Figure 13:**
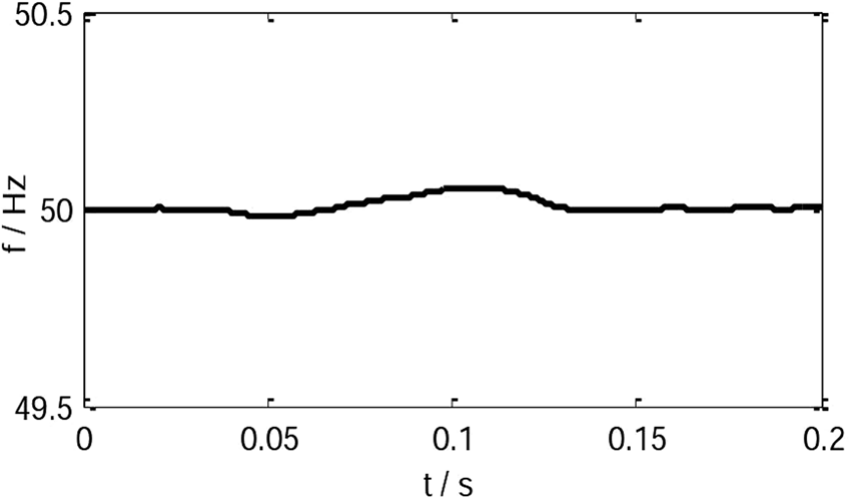
The frequency waveform of the IVSG.

When *J* = 0.2 kgm^2^ and *C* = 0.4, the frequency waveform of the IVSG is shown as Fig. 13. The frequency fluctuations always maintain within ±0.2 Hz, which meets the control requirement of frequency.

**Figure 14:**
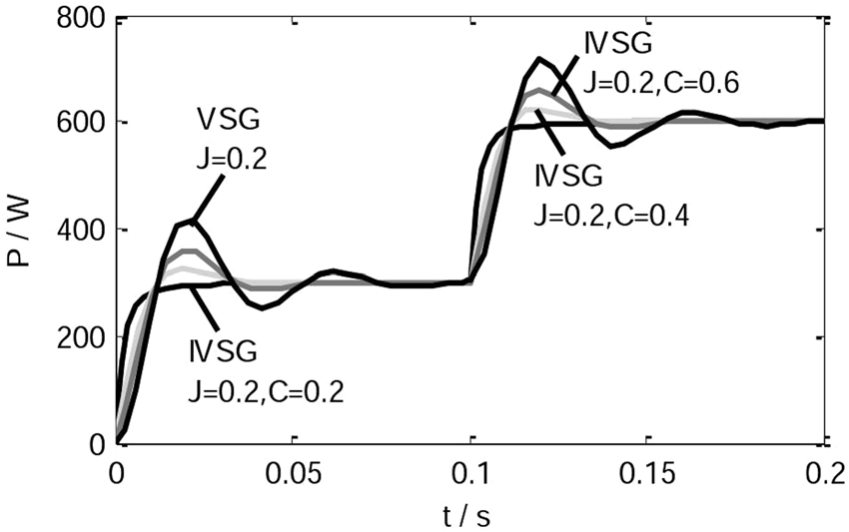
The active power waveform of the IVSG.

When *J* is 0.2 kgm^2^ and *C* takes different values, in the process of loads change, the active power waveform of the IVSG is shown in Fig. 14. As shown in Fig. 14, the introduction of the feed-forward compensation link can effectively restrain the active power fluctuation of the VSG in transient process. When the adjustable parameter *C* is set to 1, feed-forward compensation link disappears, so the effect of the IVSG control strategy is as same as the conventional VSG control strategy. Simulation result shows that properly reducing the value of adjustable parameter *C* can avoid active power overshoot and improve the response speed.

### Simulation analysis of grid connection mode

**Figure 15:**
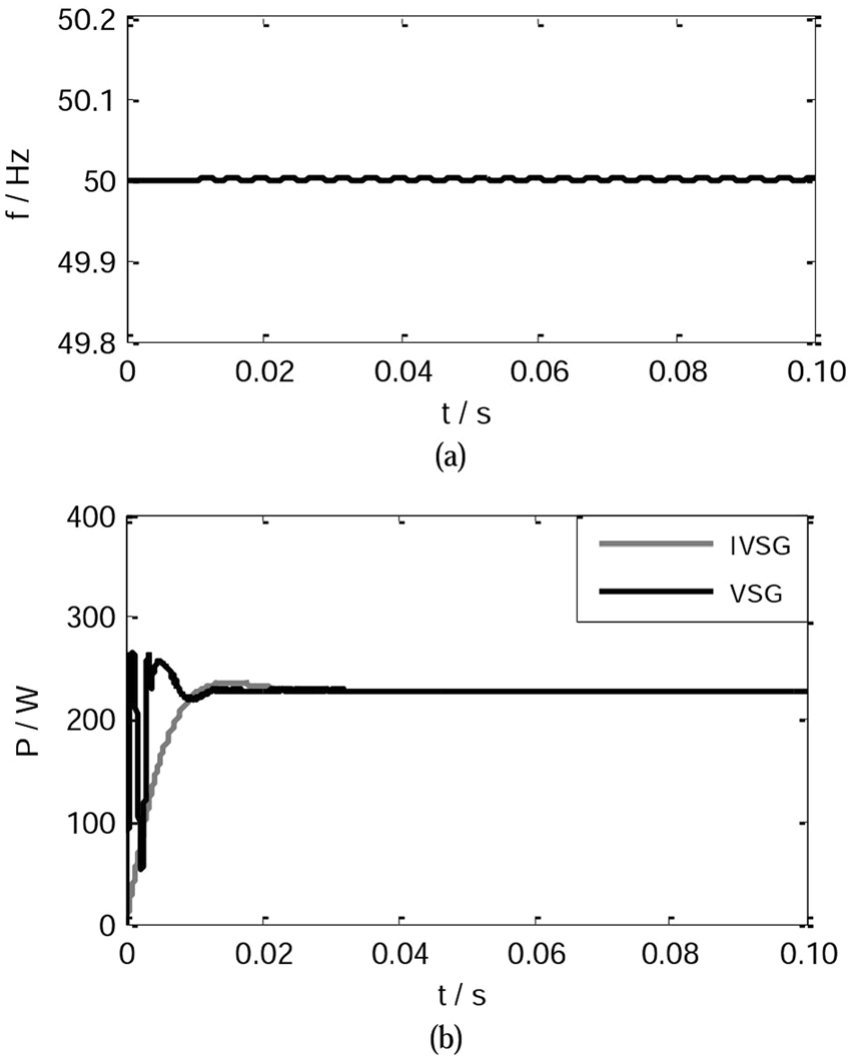
Simulation waveforms in the process of grid connection. (**a**) The frequency waveform in the process of grid connection. (**b**) The active power waveforms in the process of grid connection.

As shown in Fig. 10, the simulation time is set to 0.1 s. Initially, QF, QF_1_ and QF_2_ are turned off. When the simulation starts, QF_2_ is turned on, distributed generator is integrated into the grid. Figure 15 shows the simulation waveforms in the process of grid connection.

The frequency waveform of the IVSG is shown as Fig. 15(a), as the figure shows, the system frequency remains steady. Figure 15(b) shows the active power waveforms of the conventional VSG control strategy(*J* = 0.2 kgm^2^) and the IVSG control strategy(*J* = 0.2 kgm^2^, *C* = 0.4). As the figure shows, when the IVSG control strategy is adopted, in the process of grid connection, the fluctuation of the active power of the system is decreased significantly, and the stability of the system is improved greatly.

## Experimental results

**Figure 16:**
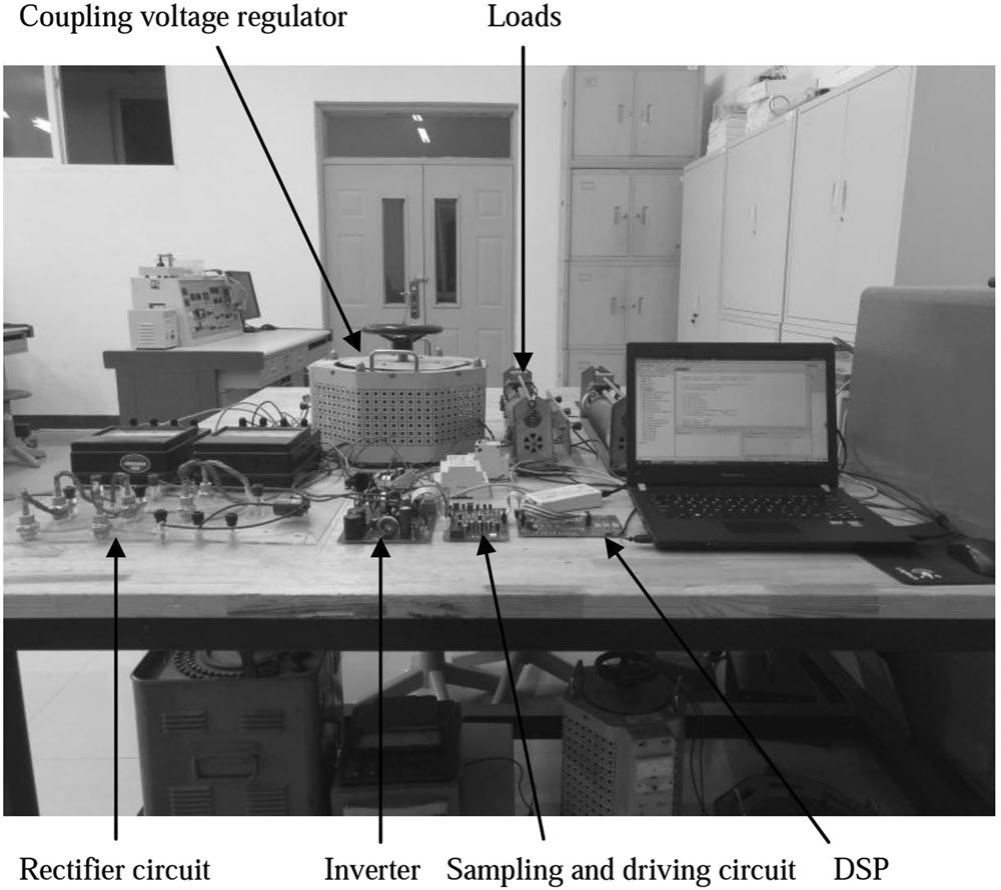
Experimental facilities.

To further prove the performance of the IVSG control strategy, the experimental platform is set up and the experiments have been carried out. Figure 16 shows the experimental facilities, experimental platform is mainly composed of uncontrolled rectifier circuit, inverter, sampling and driving circuit, controller, coupling voltage regulator and AC adjustable load. Uncontrolled rectifier circuit is used to replace the distributed energy resources. Controller takes TMS320F2812DSP as the core. A coupling voltage regulator is used to replace infinite power system. The rated capacity of the VSG is 1.5 kVA. The rated output phase voltage is 220 V/50 Hz. The value of the inertia is set to 0.2 kgm^2^. Adjustable parameter of the feed-forward compensation unit is equal to 0.4.

### Experimental results of isolated operation mode

The resistance load is 1.5 kW, and its value is adjustable. Inductive load is composed of inductance(100 mH) and coupling voltage regulator. The coupling voltage regulator is used to adjust the value of the inductive load. In the output terminal of the inverter, filter inductance is 4 mH, filter capacitance 10 μF. Parameters such as current, frequency, active power and reactive power are observed. Initially, the output active power of the inverter is 540 W, and the reactive power is 90var. 0.1 second later, active power is 1080 W, and reactive power is 180 var.

**Figure 17:**
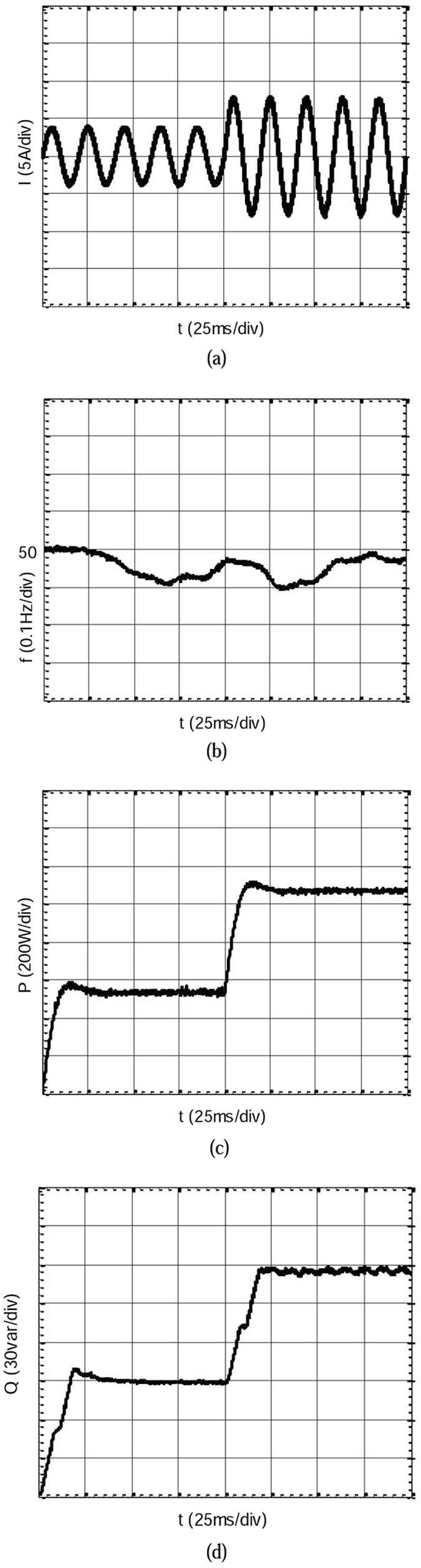
Experimental results of isolated operation mode. (**a**) The current waveform when the loads increase. (**b**) The frequency waveform when the loads increase. (**c**) The active power waveform when the loads increase. (**d**) The reactive power waveform when the loads increase.

The experimental results of isolated operation mode are shown in Fig. 17. As the figure notes, the current distortion rate of the IVSG is small, frequency fluctuations maintain within ±0.2 Hz. After a brief transient process, active power stabilizes swiftly. The power fluctuation is restrained effectively, and the overshoot of the power is small. If the adjustable parameter of the feed-forward compensation unit is further decreased, the overshoot of the active power will be accordingly decreased. The experimental results are consistent with the simulation analysis. The experiment results show that the proposed IVSG control strategy is conducive to keeping system running smoothly during the process of load changes.

### Experimental results of grid connection mode

A coupling voltage regulator is used to replace infinite power system. The rated phase voltage is 220 V/50 Hz. The value of the inertia is set to 0.2 kgm^2^. Adjustable parameter of the feed-forward compensation unit is equal to 0.4.

**Figure 18:**
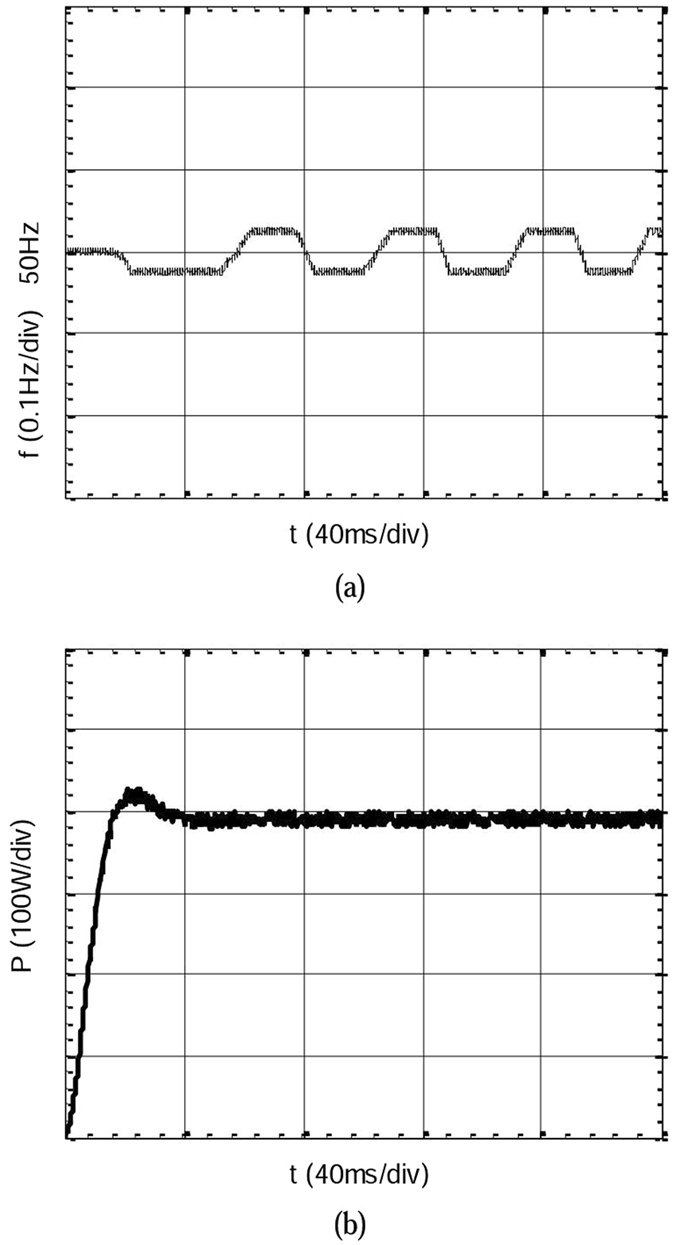
Experimental results of grid connection mode. (**a**) The frequency waveform when the grid connected. (**b**) The active power waveform when the grid connected.

The experimental results of grid connection mode are shown in Fig. 18. As the figure notes, active power tends to stabilize after a short fluctuation, the power fluctuation is restrained effectively, and the overshoot of the power is small. Frequency control works well. The experimental results are consistent with those of the simulation analysis. The experiment results show that the strategy is conducive to keeping system running smoothly during the process of grid connection.

## Conclusion

In summary, the paper proposed a distributed energy resources inverter control strategy based on IVSG. Mathematical model of the conventional VSG is studied. The closed loop transfer function of active power-frequency fluctuations for the VSG is established. Rotational inertia effect on active power in transient process is analyzed in detail. On this basis, the feed-forward compensation control unit is introduced, and the closed loop transfer function of active power-frequency fluctuations for the IVSG is constructed. To achieve the goal of reducing system order, adjustable parameter of the compensation unit can be modified. It can restrain the active power fluctuation effectively in the transient process. The idea is supported by simulation and experiment results, which indicates remarkable performance of the proposed control strategy.
